# Psycometric Power

**Published:** 1888-12

**Authors:** 


### PYSCHOMETRIC POWER.

Something odd in the way of mining has been discovered to a young Bostonian who confidently expects to make a fortune through the mysterious clairvoyance of a girl. She lives in St. Louis, but her name and exact residence are not known to the youth who has staked his money on her skill. His partner has for some years conducted mining operations successfully on the girl's advice, and has such implicit faith in her, that he voluntarily pays her and her family a liberal sum to keep her power a secret from all others.

The miner is a believer in what he calls pyschometric power. The girl, who is young and ignorant, can, on being allowed to handle an object, describe the objects which surrounded it in the place from which it was taken. This is explained by theory that everything in nature has character of its own, and impresses its influence upon all the bodies in its immediate vicinity. So the girl on being given a fragment of rock fresh from a mine is able by her peculiar sensitiveness to such influences to comprehend what was the original environment of that rock, which way the vein ran, its thickness, etc. Her power was originally discovered through her readiness in describing the furniture of a strange apartment on being given some article which had lain in the roon.

The girl's skill was recently given a test in a Colorado mine. In following up a vein of silver and gold the miners came to a "horse," a big bowlder planted across their path. In such a case it is a question with miners whether to try to go through or around the horse. In either case the chances are that they will waste a great deal of time and effort before finding the vein again. The proprieter in this dilemma got a chip of the rock which no one had handled and expressed it to his adviser in St. Louis. Back came the advice to blast away straight through the horse. In ten feet, wrote the girl, the miners would come upon a cavity containing a rich deposit, and the vein would enlarge from there on. Her advice was followed and the cavity was found just as described.

The proprietors of the mine see vast possibilities of wealth through the girl's discernment. One of them expresses the belief that thousands of people have this same psychometric power in some degree, but it requires a childlike innocence and a hermit-like freedom from ordinary cares for its free play.—*Orange Mass. Enterprise.*

